# T cell-selective deletion of Oct1 protects animals from autoimmune neuroinflammation while maintaining neurotropic pathogen response

**DOI:** 10.1186/s12974-019-1523-3

**Published:** 2019-07-03

**Authors:** Heejoo Kim, Laura Dickey, Colleen Stone, Jillian L. Jafek, Thomas E. Lane, Dean Tantin

**Affiliations:** 1 Department of Pathology, University of Utah School of Medicine, Salt Lake City, UT 84112 USA; 2 Huntsman Cancer Institute, University of Utah School of Medicine, Salt Lake City, UT 84112 USA

**Keywords:** Oct1/POU2F1, T lymphocytes, Experimental autoimmune encephalomyelitis, JHMV

## Abstract

**Background:**

Treatments for autoimmune diseases aim to dampen autoreactivity while preserving normal immune function. In CD4^+^ T cells, the transcription factor Oct1/Pou2f1 is a dispensable transcription factor for T cell development and response to primary infection, but promotes expression of target genes, including *Il2* and *Ifng*, under conditions of antigen reencounter. As a result, they are more strongly expressed upon secondary stimulation. Such repeated antigen encounters occur in memory recall responses, in autoimmunity where self-antigen can be recognized multiple times, and in chronic infection where foreign antigen is persistent. Based on these previous findings, we hypothesized that Oct1 loss would protect animals from autoimmunity but maintain normal responses to pathogens in the CNS.

**Objective:**

We used a conditional mouse *Oct1* (*Pou2f1*) allele and a CD4-Cre driver to determine the effect of T cell-specific Oct1 loss on autoimmune- and viral-induced neuroinflammation using an autoantigen-driven EAE model of autoimmunity and a JHMV model of viral infection.

**Results:**

Oct1 conditional deletion mitigated clinical scores and reduced infiltrating T cells and cytokine production in the EAE model. Consistently, Oct1-deficient CD4^+^ T cells stimulated in vitro showed increased expression of markers associated with T cell anergy, particularly in the absence of co-stimulatory signals. In contrast, anti-viral T cell effector functions are intact in the absence of Oct1, with no changes in neuroinflammation, infiltrating T cells or cytokine production.

**Conclusion:**

Our findings uncover a significant difference between the effect of Oct1 loss on autoimmune and anti-pathogen responses, which potentially could be exploited for therapeutic benefit.

**Electronic supplementary material:**

The online version of this article (10.1186/s12974-019-1523-3) contains supplementary material, which is available to authorized users.

## Introduction

Multiple sclerosis (MS) is a chronic debilitating neurological disease characterized by inflammation, demyelination, and neuronal damage caused by the inappropriate response of the host immune system towards cells of the central nervous system (CNS) [1]. Although the pathophysiology of MS is not entirely understood, active MS lesions are characterized by CNS infiltration by both CD4^+^ T cells—arranged around the periphery of active MS lesions—and CD8^+^ T cells (typically perivascular), with the subsequent activation of microglial cells, macrophages, and B cells [2]. CD4^+^ T cells can be thought of as master regulators of the immune response during MS, whereas perivascular CD8^+^ T cells, microglial cells, macrophages, and even neutrophils largely mediate white matter damage [2]. Genome-wide association studies (GWAS) pinpoint the major histocompatibility complex (MHC) genes located in the human leukocyte antigen (HLA) region as having the strongest influence on disease, further emphasizing the importance of T cells in MS pathophysiology [3].

Oct1/Pou2f1 is a POU-domain transcription factor that in mice is dispensable for T cell development and response to primary infection but is important for the formation of CD4^+^ central memory cells [4]. Consequently, CD4^+^ T cells lacking Oct1 are completely defective in memory recall responses. Memory T cells are highly prone to making proinflammatory cytokines, and memory or memory-like cells can underlie autoimmunity (including T1D), even in cases of persistent self-antigen exposure [5–7]. In vitro, Oct1 and its cofactor OCA-B coordinately control a large cohort of critical direct target genes in CD4^+^ lymphocytes, including *Il2*, *Il21*, *Stat5a*, *Ifng*, *Tbx21* (*Tbet*), *Csf2* (*Gmcsf*), *Tnfrsf4* (*Ox40*), *Icos*, and *Ctla4* [4]. Interestingly, Oct1 and OCA-B are dispensable for the baseline activity of these genes. For example, CD4^+^ T cells lacking Oct1 due to germline or conditional deletion develop normally and express normal levels of the key T cell effector cytokine gene IL-2 upon primary stimulation [4, 8]. Instead, Oct1 and OCA-B strongly regulate these genes under conditions of antigen re-encounter such that secondary stimulation of resting but previously activated cells results in expression defects of 20-fold or more [8]. During CD4^+^ T cell polarization, Oct1 works together with another transcription factor, CTCF, to mediate physical communication between the *Il4*, *Ifng*, and *Il17a* target loci [9]. The Oct1 cofactor OCA-B/Bob.1 has also been linked to CD4^+^ central memory cell formation and function and to the formation of Th17 cells [4, 10]. Cumulatively, the findings point to a potent role of Oct1 and OCA-B in the control of CD4^+^ T cell responses, but only under specific circumstances involving repeated antigen exposure. This normal development and stimulation response forms part of a potential “therapeutic window” in which targeting Oct1 and its associated pathways could be used to treat autoimmune responses while sparing normal immune function.

In addition to immune memory, repeated antigen encounter also occurs in situations such as chronic infection, graft-versus-host disease, tumor immunity, and autoimmunity. In the case of the latter, human GWAS studies show strong associations between polymorphisms in binding sites for Oct1 and predisposition for autoimmune disease including rheumatoid arthritis, celiac disease, type-1 diabetes, ulcerative colitis, autoimmune thyroiditis, and MS [11–14]. The strong associations with processes governing neuroinflammatory disease, and MS in particular, lead us to consider the role of Oct1 in neuroinflammatory T cell responses to autoantigens and viral infection.

Here, we show that Oct1 loss in T cells greatly attenuates clinical responses, T cell infiltration, and cytokine production in a murine experimental autoimmune encephalomyelitis (EAE) model, while maintaining immune responses to JHMV infection. EAE is auto-antigen-driven and is the prototypic mouse model of MS. The decreased clinical responsiveness was associated with changes in the expression of anergy-associated surface proteins on CD4^+^ T cells upon stimulation in vitro, in particular in the absence of co-stimulatory signals. Using a model of neuroinflammation induced by intracranial infection by the neurotropic JHM strain of mouse hepatitis virus (JHMV), we observed few differences in clinical scores, infiltrating T cells and macrophages and cytokine expression. Viral clearance was slowed but complete in animals with Oct1-deficient T cells. Cumulatively, these results suggest that targeting pathways involving Oct1 in CD4^+^ T cells may provide a novel therapeutic avenue for the treatment of MS and other neuroinflammatory diseases, while largely sparing beneficial immune function.

## Material and methods

### Laboratory mice

All mice used in this study were on the C57BL/6 J strain background. *Oct1* (*Pou2f1*) conditional mice crossed to CD4-Cre have been previously described [4]. All animal experiments were approved by the University of Utah Institutional Animal Care and Use Committee (17-05008).

### Induction and scoring of EAE

EAE was initiated using a myosin oligodendrocyte protein (MOG)/*Bordetella pertussis* toxin (PT) method [15]. Briefly, mice were subcutaneously injected with 0.2 μmol of MOG_35-55_ peptide (MEVGWYRSPFSRVVHLYRNGK, synthesized at the University of Utah HSC Core) in complete Freund’s adjuvant (CFA, Sigma, 2 mg/mL). Two hundred nanograms of PT (Sigma) was injected into the mice twice intravenously. Clinical scores were determined based on the following criteria: 0, no clinical disease; 1, loss of tail tonicity; 2, mild hind limb paresis; 3, moderate hind limb paralysis; 4, paraplegia; 5, quadriplegia, coma, or death. For tissue analysis, animals were sacrificed at peak disease (days 20–21).

### Leukocyte isolation and intracellular cytokine staining

Leukocytes were isolated from spinal cords and cervical lymph nodes using a Percoll gradient method [16–18]. Briefly, tissues were dissociated by grinding and passed through a nylon strainer. Cells were centrifuged with 80% and 40% Percoll at 1300×*g* at room temperature. Cells at the interface between 40 and 80% Percoll were taken. For intracellular staining, isolated cells were stimulated with PMA (Sigma, 50 ng/mL) and ionomycin (Sigma, 1 μg/mL) along with brefeldin A (Golgi Plug, Becton-Dickenson) for 4 h and were fixed with cell fixation/permeabilization solution (BD Cytofix/Cytoperm^tm^) according to manufacturer’s protocol. Antibodies used for flow cytometry were as follows: FITC conjugated anti-mouse CD4 (Biolegend), PerCP conjugated anti-mouse CD8a, APC-conjugated anti-mouse IFNγ, and PE-conjugated anti-mouse IL-17 (eBioscience).

### In vitro culture

Spleens were harvested from CD4-Cre;*Oct1*^*fl/fl*^ and control CD4-Cre animals 10 days after inoculation with MOG_35–55_ peptide and CFA. Single-cell suspensions were prepared by grinding spleens through 70-μm strainers. CD4^+^ T cells were isolated by a mouse CD4^+^ T cell isolation kit (Miltenyi Biotec). The isolated CD4^+^ T cells were cultured as described previously [8] and stimulated with 5 μg/ml plate-bound anti-CD3ε (BD Bioscience) and 2 μg/ml anti-CD28 antibodies (eBioscience) for 24 h.

### JHMV

For intracranial (i.c.) injections, age-matched (5–7 weeks) C57BL/6 mice of different genotypes were anesthetized with an intraperitoneal (i.p.) injection of 200 μL of a mixture of ketamine (Hospira, Lake Forest, IL, USA) and xylazine (Phoenix Pharmaceutical, Saint Joseph, MO, USA) in Hank’s balanced salt solution (HBSS). Mice were injected i.c. with 200 plaque-forming units (PFU) of JHMV (strain V34) suspended in 30 μL HBSS. Clinical severity was assessed using a previously described 4-point scoring scale [19]. For analysis of viral titers, mice were sacrificed at indicated time points. One half of each brain was homogenized and used in a plaque assay performed using the DBT mouse astrocytoma cell line [19].

### Cell isolation and flow cytometry

Immunophenotyping of immune cells present within brains and spinal cords of JHMV-infected mice at defined times post-infection (p.i.) was accomplished by homogenizing isolated tissue and generating single-cell suspensions for analysis by flow cytometry using previously described procedures [19–21]. In brief, isolated cells were stained with the following antibodies: APC-conjugated rat anti-mouse CD4 and a PE-conjugated tetramer specific for the CD4 immunodominant epitope present within the JHMV matrix (M) glycoprotein spanning amino acids 133-147 (M133-147 tetramer) to determine total and virus-specific CD4^+^ cells, respectively [19–21]; APC-conjugated rat anti-mouse CD8a and a PE-conjugated tetramer specific for the CD8 immunodominant epitope present in the spike (S) glycoprotein spanning amino acids 510-518 (S510518) to identify total and virus-specific CD8^+^ cells, respectively [19–21]. Tetramers were synthesized by the NIH tetramer core facility: APC-conjugated rat anti-mouse CD4 and PE-conjugated anti-CD25 to determine total T-regulatory cells and BV510-conjugated rat anti-mouse CD45 and FITC-conjugated anti-F4/80 to identify macrophages. Samples were analyzed using a BD LSR Fortessa X-20 flow cytometer and FlowJo software.

### Histology

Spinal cords were isolated at defined time points and fixed overnight with 4% paraformaldehyde at 4 °C. Sections were subsequently cryoprotected in 30% sucrose for 5–7 days, separated into 12 coronal sections and embedded in optimum cutting temperature (OCT) formulation (VWR, Radnor, PA, USA) [15, 19–21]. Coronal sections (8-μm thick) were cut, and sections were stained with luxol fast blue (LFB) in combination with hematoxylin and eosin (H&E). Areas of total white matter and demyelinated white matter were determined with Image J Software. The percent demyelination was calculated by dividing the area of demyelinated white matter by the total white matter area using established methods previously described [19].

### Statistical analysis

All error bars denote ± SEM. Student *t* tests were used to ascribe statistical significance. For all figures, * = *p* value ≤ 0.05 and ** = *p* value ≤ 0.01.

## Results

**Fig. 1:**
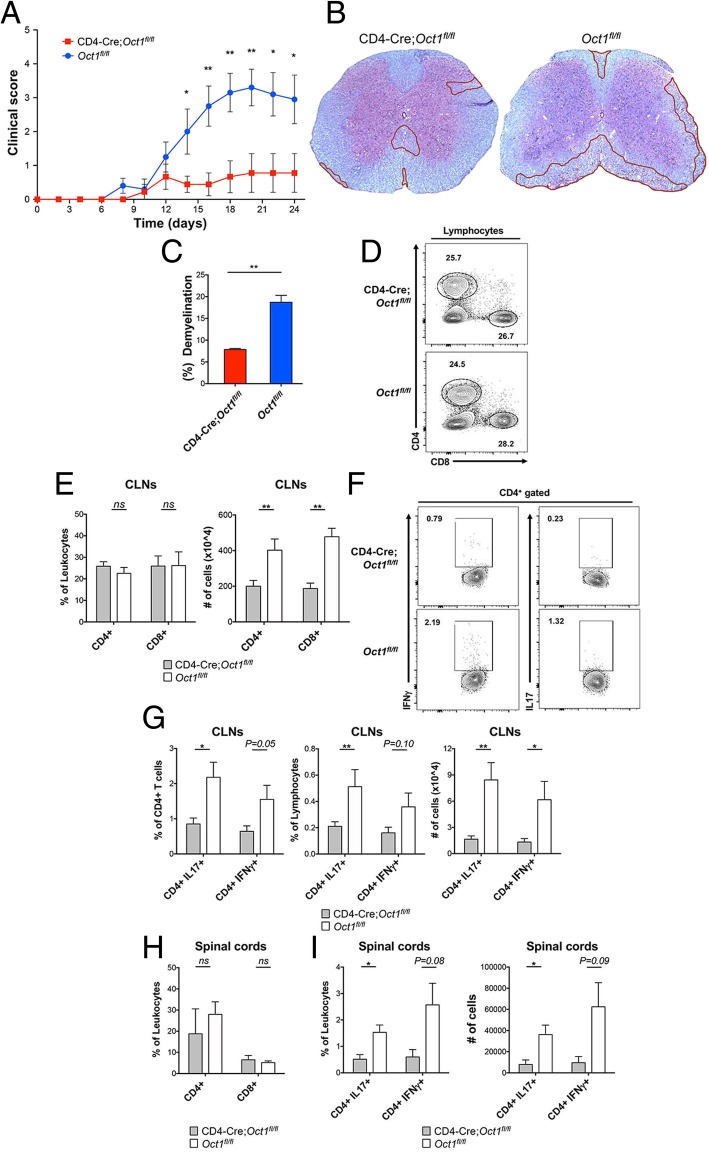
Loss of Oct1 in T cells protects mice using an EAE model of MS. **a** CD4-Cre;*Oct1*^*fl/fl*^ (*n* = 9) or *Oct1*^*fl/fl*^ (*n* = 10) mice were injected with MOG_35–55_ peptide and pertussis toxin to generate EAE. Clinical scores were determined during the post-treatment timecourse. **b** Representative LFB staining of thoracic spinal cord sections from animals taken at peak disease (day 21). Areas of demyelination are outlined in red. **c** Quantification of demyelination in experimental mice. Mean % demyelination from six sections of two mice. **d** Cervical lymph node lymphocytes were isolated from EAE-induced CD4-Cre;*Oct1*^*fl/fl*^ (*n* = 6) or *Oct1*^*fl/fl*^ (*n* = 6) mice and analyzed by flow cytometry. Frequencies of CD4 and CD8 cells from representative animals are shown. **e** Mean CD4^+^ and CD8^+^ T cell percentages (left panel) and total cell numbers (right panel). Cells were independently purified from the CLNs of six separate six mice. **f** Representative data showing frequencies of cytokine-producing CD4^+^ cells in the CLN. **g** Percentages (left and middle panels) or total cell numbers (right panel) of cytokine-producing CD4^+^ T cells are plotted. *N* = 6 for each group. Mean of results is shown. **h** Mean CD4^+^ and CD8^+^ T cell percentages in the spinal cords. *N* = 3–4 for each group. **i** Cytokine-producing CD4^+^ T cell percentages (left panel) and total cell numbers (right panel). Cells were independently purified from the spinal cords of separate six mice

To determine the effects of Oct1 in T cells on the pathogenesis of a neuroautoimmune disease, we used Oct1 T cell conditional mice (CD4-Cre;*Oct1*^*fl/fl*^ [4] and control mice (*Oct1*^*fl/fl*^) in conjunction with a MOG-EAE model of MS. Following inoculation with peptide corresponding to myelin oligodendrocyte glycoprotein (MOG_35–55_, see methods) and with Freund’s complete adjuvant, pertussis toxin was injected into mice to increase blood-brain barrier permeability. Disease severity was determined by evaluating the clinical score. C57BL/6 mice develop clinical symptoms 9–14 days after MOG injection [22]. As shown in Fig. 1a, CD4-Cre;*Oct1*^*fl/fl*^ mice were significantly protected, with clinical scores less than 1, while the control *Oct1*^*fl/fl*^ mice exhibited much higher clinical involvement at the peak point of disease (day 20, Fig. 1a). Additionally, we collected spinal cords for histopathological scoring 21 days after EAE induction. The degree of demyelination in control mice was doubled to that of the CD4-Cre;*Oct1*^*fl/fl*^ group (Fig. 1b, c). These results reveal that Oct1 deletion in T cells strongly protects mice from clinical symptoms in a MOG-EAE model of MS.

T cells are indispensable for the pathogenesis of EAE and MS [23]. IFNγ and IL-17 expression in CNS-infiltrating Th1 and Th17 CD4^+^ T cells in EAE correlates with clinical severity [23–25]. Both CD4^+^ and CD8^+^ T cells contribute to clinical and histologic disease. CD8^+^ T cells are recruited to lesions and mediate the destruction of oligodendrocytes and axons [26, 27]. Therefore, we screened T cell populations in the draining cervical lymph nodes (CLNs) and in the spinal cords of Oct1 conditional and control mice at the peak of disease progression to determine if T cells lacking Oct1 have reduced autoimmune activity in the CNS and CLNs. Although the percentages of CLN CD4^+^ and CD8^+^ were similar between the groups (Fig. 1d, e left panel), fewer (*p* < 0.01) total CD4^+^ and CD8^+^ T cells were detected in the CD4-Cre;*Oct1*^*fl/fl*^ group compared to control *Oct1*^*fl/fl*^ mice (Fig. 1e, right panel). This result is suggestive of reduced lymph node cellularity in the EAE model. Because CD4^+^ T cells are the primary inducers in EAE models [23], we also profiled cytokine production in these cells. Both frequencies and total numbers of IL-17- and IFNγ-producing CD4^+^ T cells were reduced in the CLNs of CD4-Cre;*Oct1*^*fl/fl*^ mice compared to *Oct1*^*fl/fl*^ controls (Fig. 1f, g). As with CLNs, frequencies of CD4^+^ and CD8^+^ T cells were similar in the spinal cords of CD4-Cre;*Oct1*^*fl/fl*^ and *Oct1*^*fl/fl*^ mice (Fig. 1h). Total numbers of CD4^+^ and CD8^+^ T cells were also similar between CD4-Cre;*Oct1*^*fl/fl*^ and *Oct1*^*fl/fl*^ mice (not shown). However, as in the CLNs, proinflammatory cytokine production was strongly reduced in the infiltrating T cells in the spinal cords of CD4-Cre;*Oct1*^*fl/fl*^ mice compared to controls (Fig. 1i). These data indicate that loss of Oct1 in T cells strongly protects animals from clinical symptoms of EAE and that this protection is associated with decreased CNS T cell proinflammatory cytokine expression.

T cell anergy is a peripheral tolerance mechanism induced by TCR stimulation in the absence of co-stimulatory signals, in which T cells become poorly reactive, protecting animals from potential autoreactivity [28–31]. Oct1 loss results in decreased expression of target genes such as *Ifng*, selectively in the context of repeated T cell stimulation [8]. Gene expression profiling using CD4^+^ T cells deficient in the Oct1 cofactor OCA-B reveals not only that these genes are downregulated, but also identifies increases in the expression of genes associated with anergy, e.g., *Ctla4* [4]. Anergic responses can be modeled in vitro by providing T cells with primary TCR simulation (via immobilized anti-CD3ε monoclonal antibodies) in the absence of co-stimulation [31]. To determine the effect of Oct1 loss on anergic responses, we harvested CD4^+^CD44^+^ T cells (consisting mostly of pre-activated resting cells) from the spleens of CD4-Cre;*Oct1*^*fl/fl*^ and control *Oct1*^*fl/fl*^ animals, restimulated them for 24 h ex vivo using anti-CD3 antibodies with or without CD28 co-stimulation, and profiled the expression of proteins associated with activation and anergy.

**Fig. 2:**
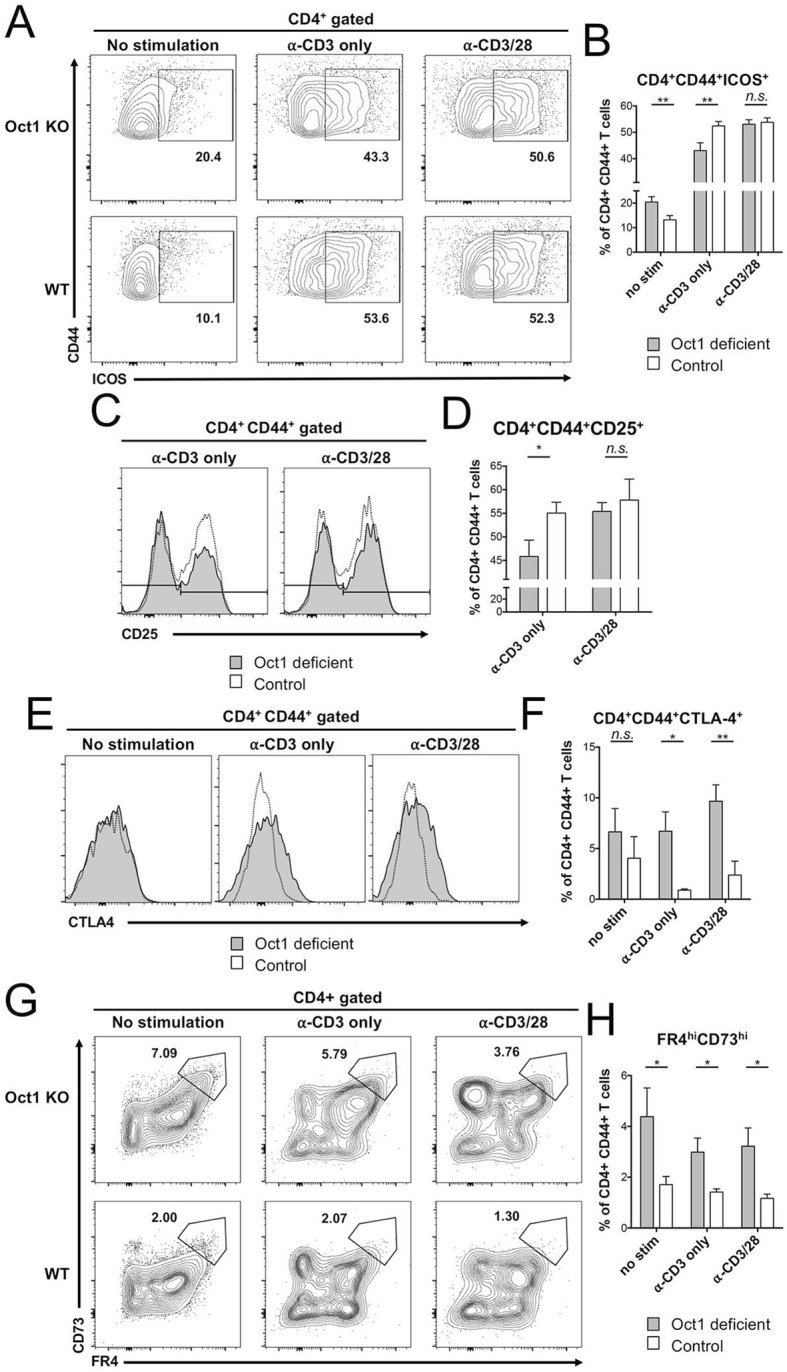
In vitro stimulation of T cells lacking Oct1 results in decreased expression of markers associated with activation and increased expression of markers associated with anergy. **a** Oct1-deficient and control CD4^+^ T cells stimulated in vitro with indicated antibodies and analyzed by flow cytometry. Representative frequencies of ICOS-expressing CD4^+^ CD44^+^ cells are shown. **b** Quantification of cells independently purified from the spleens of three mice, with three technical culture replicates for each mouse. **c** Representative flow cytometry plots showing frequencies of CD25-expressing CD4^+^ CD44^+^ cells in Oct1-deficient and control CD4^+^ T cells. **d** Quantification from three animals. **e** Representative expression of CTLA-4 in CD4^+^ CD44^+^ T cells is plotted as histograms for Oct1-deficient and control CD4^+^ T cells. **f** CTLA4^+^ percentages from three animals, with three culture replicates per animal, are plotted. **g** Expression of FR4 and CD73 in Oct1-deficient and control CD4^+^CD44^+^ cells. **h** Averaged percentages of FR4^hi^CD73^hi^CD4^+^CD44^+^ cells are plotted

ICOS (inducible T cell costimulator) has an important but complex role in the induction of T cell anergy in vitro and the development of autoimmunity in vivo [32–34]. Analyzing ICOS expression by flow cytometry, we found that baseline ICOS levels were ~ 2-fold higher in naïve Oct1-deficient cells but that no differences were apparent in fully stimulated cells receiving anti-CD3/28 (Fig. 2a, b). Interestingly, stimulating Oct1-deficient cells with anti-CD3 alone resulted in significantly reduced ICOS expression in Oct1-deficient cells compared to the control group (Fig. 2a, b). Both the increased baseline ICOS expression in resting cells and decreased expression upon anergic stimulation are consistent with the observed protection in an EAE model, as ICOS blockade during antigen priming (days 1–10) increases brain inflammation and promotes EAE, whereas blockade later in EAE pathogenesis (days 9–20) decreases CNS leukocyte infiltration and is protective [33, 35]. Additionally, the expression of CD25, the high-affinity IL-2 receptor induced upon CD4^+^ T cell activation [36], was decreased in Oct1-deficient cells specifically in anergic conditions lacking co-stimulation. T regulatory cells (Tregs) express CD25 and also produce IL-10; however, IL-10 production in these cells was similar to control CD4+ T cells (Additional file 1), indicating that these in vitro differences are associated with effector T cells. In contrast to CD25, CD44 expression levels were similar between the groups (Fig. 2c, d). These results show that ICOS and CD25 levels are altered in Oct1-deficient cells in a manner consistent with observed protection in the EAE model.

We also analyzed the expression of inhibitory molecules correlated with anergic responses. The expression of CTLA4, a checkpoint inhibitor and an anergy marker [37], was increased by Oct1 loss in both anergic and full activation conditions (Fig. 2e, f). Similarly, FR4/CD73 double-positive cell frequencies were increased in Oct1-deficient cells compared to control cells in all conditions (Fig. 2g, h)**.** CD4^+^CD44^+^FR4^hi^CD73^hi^ cells are associated with anergy [30, 38]. Frequencies of IFNγ or IL-17 producing cells in Th1 or Th17 differentiating culture conditions were also measured to investigate if Oct1 loss affects cytokine expression levels. No differences were observed after 5 days of stimulation with MOG_35–55_ peptide (data not shown). Thus, in vitro stimulation of T cells lacking Oct1 results in decreased expression of surface proteins associated with activation and increased expression of proteins associated with anergy.

**Fig. 3:**
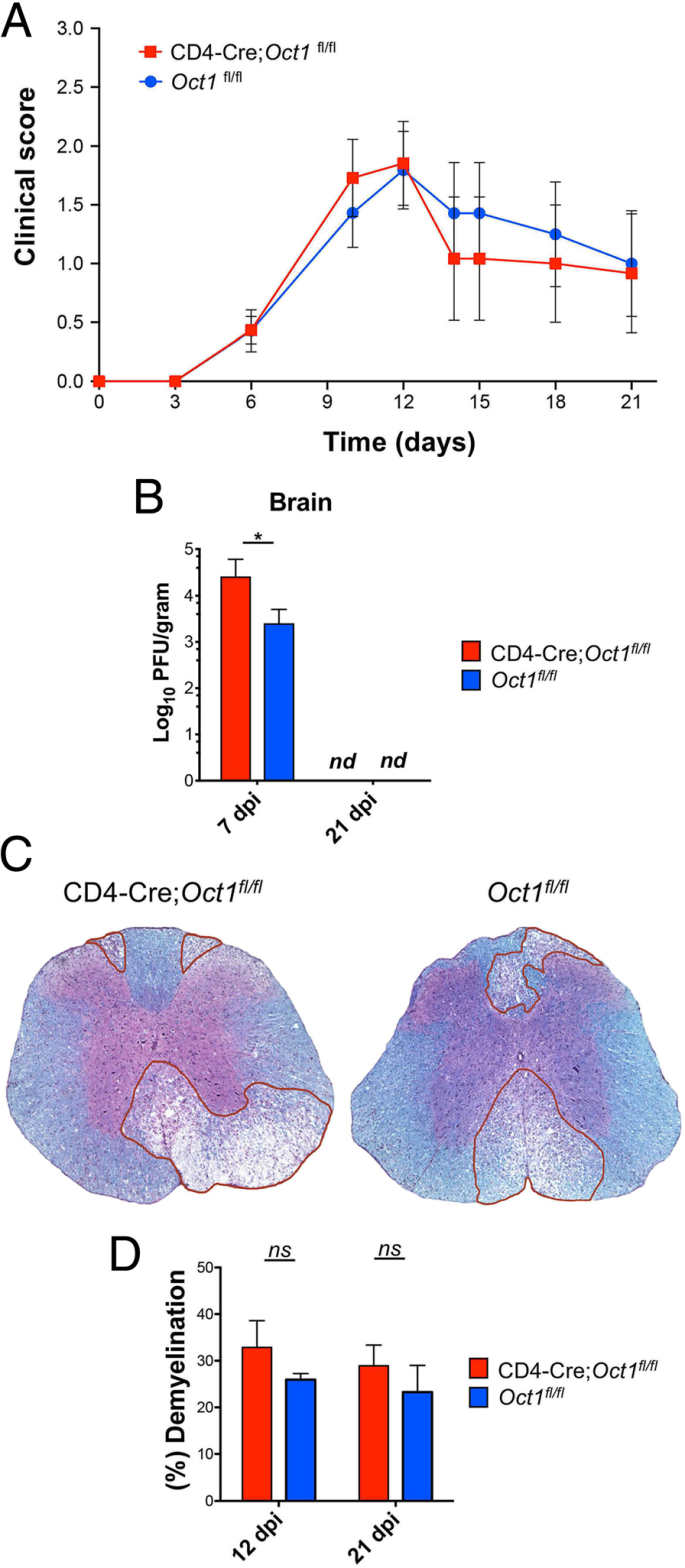
The absence of Oct1 in T cells does not impact disease in JHMV-infected mice. **a** CD4-Cre;*Oct1*^*fl/fl*^ (*n* = 15) or *Oct1*^*fl/fl*^ mice (*n* = 14) were infected i.c. with 200 PFU of JHMV and disease severity assessed. Clinical disease was recorded to day 21 p.i. **b** Brain viral titers were determined at days 7 and 21 p.i., (n.d., not detected). **c** Representative LFB stained thoracic spinal cord sections from experimental mice at day 12 p.i. **d** Quantification of average demyelination from CD4-Cre;*Oct1*^*fl/fl*^ (*n* = 4, 12 dpi; *n* = 3, 21dpi) and *Oct1*^*fl/fl*^ mice (*n* = 3, 12 dpi; *n* = 5, 12 dpi) at days 12 and 21 p.i

**Fig. 4:**
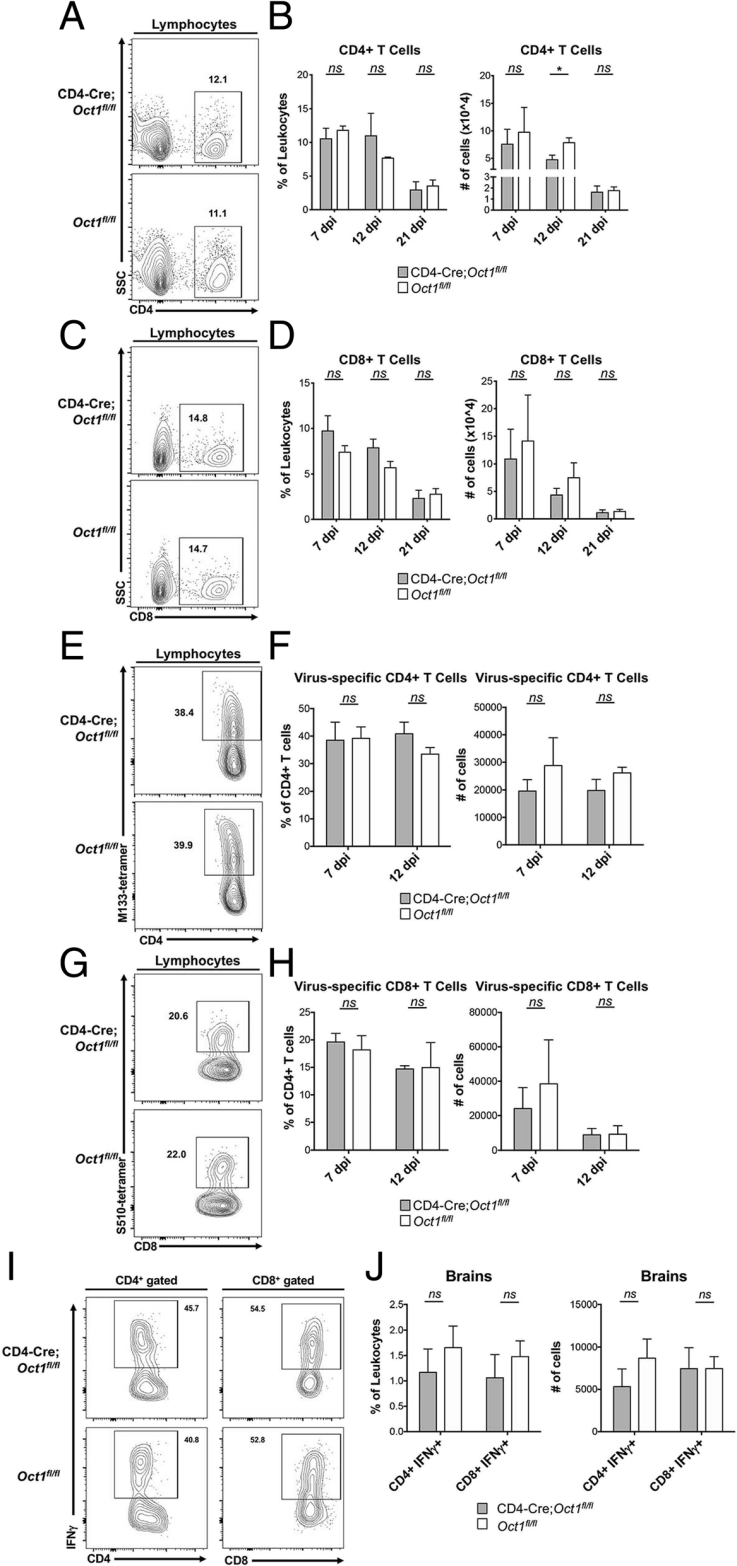
Normal immune responses in Oct1 T cell-deficient mice during JHMV infection. **a** CD4-Cre;*Oct1*^*fl/fl*^ or *Oct1*^*fl/fl*^ mice were infected i.c. with 200 PFU of JHMV and sacrificed at days 7 (*n* = 8), 12 (*n* = 4–5), and 21 (*n* = 6) p.i. to assess T cell infiltration into the brain. Representative flow analysis depicting CD4^+^ T cell infiltration into brains of mice at day 7 p.i. **b** Quantification of CD4^+^ T cells as shown by calculating both frequencies and numbers of isolated cells. **c** Representative flow analysis depicting CD8^+^ T cell infiltration into brains of mice at day 7 p.i. **d** Quantification of CD8^+^ T cells as shown by calculating both frequencies and numbers of isolated cells. **e** Representative M133-147 tetramer staining of CD4^+^ T cells from brains of JHMV-infected experimental mice. **f** Quantification of frequency and numbers of M133-147 tetramer CD4^+^ T cells from experimental groups. **g** Representative S510-518 tetramer staining of CD8^+^ T cells from brains of JHMV-infected experimental mice. **h** Quantification of frequency and numbers of M133-147 tetramer CD4^+^ T cells from experimental groups. Data presented are derived from two independent experiments; day 7 p.i., CD4-Cre;*Oct1*^*fl/fl*^ *n* = 8,*Oct1*^*fl/fl*^ mice *n* = 8; day 12 p.i., CD4-Cre;*Oct1*^*fl/fl*^ *n* = 5,*Oct1*^*fl/fl*^ mice *n* = 4. **i** IFNγ-producing CD4^+^ (left panel) and CD8^+^ (right panel) CNS-infiltrating T cell percentages are shown for representative animals. **j** Averaged frequencies (left panel) and total cell numbers (right panel) of CD4^+^ and CD8^+^ cells analyzed as in **b**. *N* = 6 for each group

The above findings suggest that targeting Oct1 could be a viable therapeutic strategy for MS. Prior findings using acute infection with the model pathogen LCMV indicate that Oct1 in T cells is dispensable for pathogen response and clearance, but necessary for robust memory recall responses [4]. However, the role of Oct1 in neuroinflammation caused by neurotropic viruses has not been tested. Significantly increased pathology in the case of JHMV would suggest that targeting Oct1 directly as a treatment for autoimmunity will result in unwanted side effects. To determine whether Oct1 mediates disease severity in viral-induced encephalomyelitis, we studied responses to JHMV. JHMV is a glial-tropic coronavirus and well-accepted model of viral-induced encephalomyelitis and immune-mediated demyelination [39–41]. Intracranial inoculation of C57BL/6 mice with JHMV typically results in acute encephalomyelitis, immune-mediated demyelination, and hind limb paralysis. T cell responses are critical for controlling JHMV replication within the CNS [42]. Age-matched *Oct1*^*fl/fl*^ and CD4-Cre;*Oct1*^*fl/fl*^ mice were intracranially (i.c.) inoculated with JHMV (200 PFU), and the severity of clinical disease and survival were monitored. JHMV-infected CD4-Cre;*Oct1*^*fl/fl*^ mice demonstrated no differences in clinical disease severity out to 21 days (Fig. 3a). Viral titers in the brains of JHMV-infected CD4-Cre;*Oct1*^*fl/fl*^ compared to control mice were studied at defined times p.i. Although viral titers were elevated in the CNS at day 7 p.i. in CD4-Cre;*Oct1*^*fl/fl*^ mice compared to control animals, by day 21, p.i. viral titers were below the level of detection (~ 100 PFU/g tissue) in both groups (Fig. 3b). This result indicates that viral clearance is intact in Oct1 T cell-deficient mice. Supporting these findings, we observed similar degrees of demyelination at peak disease (day 12 p.i.) and endpoint (day 21 p.i., Fig. 3c, d). Numbers of infiltrating CD4^+^ (Fig. 4a, b) or CD8^+^ T cells (Fig. 4c, d) in CD4-Cre*;Oct1*^*fl/fl*^ mice compared to control mice were similar. Furthermore, using tetramer staining [19–21], we observed no significant differences in virus-specific CD4^+^ T cells (Fig. 4e, f) or CD8^+^ T cells (Fig. 4g, h). There was also no difference in the percentage of CD25^+^ cells or macrophage accumulation within the CNS of JHMV-infected mice at any time point (data not shown). We studied proinflammatory cytokine expression in CNS T cells from animals euthanized 21 days p.i. We did not observe significant levels of IL-17 expression (not shown), consistent with prior findings that Th17 cells do not play a prominent role in this model [43, 44]. IFNγ-expressing cells were present; however, we observed no significant differences in either percentage or numbers, or the degree of IFNγ production (Fig. 4i, j). Together, these data suggest that Oct1 loss in T cells does not impact neurologic disease or immune-mediated demyelination or T cell functionality in response to JHMV infection of the CNS.

## Discussion

Here, we show that expression of the transcription factor Oct1 in T cells promotes CNS autoimmunity using MOG-EAE models, but only minimally participates in CNS anti-viral immunity. These results suggest that targeting Oct1, and its associated activities and pathways, could be used to treat autoimmunity while sparing viral pathogen-directed immune function.

Oct1 mechanisms of transcription regulation have been studied in CD4^+^ T cells [4, 8]. Direct target genes include *Il2*, *Ifng*, *Csf2* (*Gmcsf*), *Icos*, and *Ctla4*. However, unlike NF-AT or AP-1, Oct1 is dispensable for the baseline activity of these genes. Stimulation of primary CD4^+^ Oct1-deficient naïve T cells results in normal levels and induction kinetics of the key T cell effector cytokine IL-2 [4, 8]. The normal T cell development and response to initial stimulation forms part of a potential “therapeutic window” in which targeting Oct1 and its associated pathways could be used to treat autoimmune responses with minimal side effects. Instead, Oct1 target genes show severely defective expression (100-fold or more) upon a second encounter with antigen and co-stimulatory signals [4, 8]. In vivo, CD4^+^ T cells lacking Oct1 mount a normal response to the acute viral pathogen LCMV, but fail to form memory cells in appreciable numbers. Those memory cells that are formed are defective in pathogen recall responses [4]. Memory cells are the most prone to making proinflammatory cytokines, and memory or memory-like cells can underlie autoimmunity, even in cases of persistent self-antigen exposure [5–7]. These findings, and the strong associations between human polymorphisms in binding sites for Oct1 and predisposition for autoimmune disease including MS [11–14], suggested a possible role for Oct1 in promoting MS.

MOG-EAE is an established model of MS, driven by inoculation with autoantigen in the presence of proinflammatory signals. Using this model, we showed that loss of Oct1 in T cells protects animals from clinical symptoms of EAE. This protection was associated with decreased CLN lymphadenopathy and proinflammatory cytokine expression, as well as decreased CNS T cell infiltration and cytokine expression.

T cell tolerance can be induced centrally, through thymic selection, or peripherally, due to the activity of induced Tregs or induction of anergy [28, 45]. We found that stimulation of CD4^+^ T cells lacking Oct1 with CD3 alone, to mimic TCR stimulation in the absence of co-stimulatory signals, significantly increased signs of anergy compared to control Oct1-sufficient cells. Decreased ICOS and CD25 levels were observed in Oct1-deficient cells in the absence of co-stimulation, whereas no differences were observed with co-stimulation. ICOS is a co-stimulatory molecule expressed by activated T cells with an important but complex role in the induction of T cell anergy in vitro and the development of autoimmunity in vivo [32–34]. Blocking ICOS during antigen priming promotes EAE, whereas blocking ICOS later in the disease course is protective [33, 35]. Interestingly, in addition to the decreased ICOS levels observed upon anergic stimulation, we found that unstimulated CD4^+^ T cells lacking Oct1 expressed baseline ICOS at higher levels. Both observations are therefore consistent with the observed protection in EAE models. Oct1-deficient cells also showed higher levels of the inhibitory receptor CTLA-4 and the anergic markers CD73 and FR4.

Our findings also reveal that Oct1 is dispensable for clinical responses to JHMV-induced neurologic disease, as clinical scores and demyelination were superimposable in this model. Expression of Oct1 accelerated but was not necessary for effective viral control. However, total and antiviral T cell numbers, cytokine expression, and macrophage recruitment were broadly unaffected. Examples include T cell percentages as well as numbers of cytokine-expressing cells and regulatory T cells.

## Conclusion

Collectively, these results indicate that while Oct1 loss has only modest effects on viral-induced inflammation, it profoundly improves responses to autoantigen-driven disease. These results suggest that targeting Oct1 and its associated upstream and downstream pathways (such as the cofactor OCA-B) may be of therapeutic benefit in autoimmunity while sparing viral pathogen-directed immune function. Additional work will be required to identify which components of this pathway are potentially targetable.

## Additional file

Additional file 1: **Figure S1** IL-10 expression is unchanged in Oct1-deficient T cells expressing decreased levels of CD25. Cells were prepared identically to Fig. 2. (JPG 239 kb)

## Data Availability

Please contact the author for data requests.

## Abbreviations

CFA: Complete Freund’s adjuvant
CLNs: Cervical lymph nodes
CNS: Central nervous system
EAE: Experimental autoimmune encephalomyelitis
HBSS: Hank’s balanced salt solution
i.c: Intracranial
i.p: Intraperitoneal
ICOS: Inducible T cell costimulator
JHMV: JHM strain of mouse hepatitis virus
LFB: Luxol fast blue
MOG: Myosin oligodendrocyte protein
MS: Multiple sclerosis
OCT: Optimum cutting temperature
p.i: Post-infection
PFU: Plaque-forming units
PT: *Bordetella pertussis* toxin
Treg: T regulatory cell
