# Supplementary figures and images for: T cell-selective deletion of Oct1 protects animals from autoimmune neuroinflammation while maintaining neurotropic pathogen response
Source: J Neuroinflammation. 2019 Jul 3;16:133. doi: 10.1186/s12974-019-1523-3 (PMC6607600; doi:10.1186/s12974-019-1523-3)

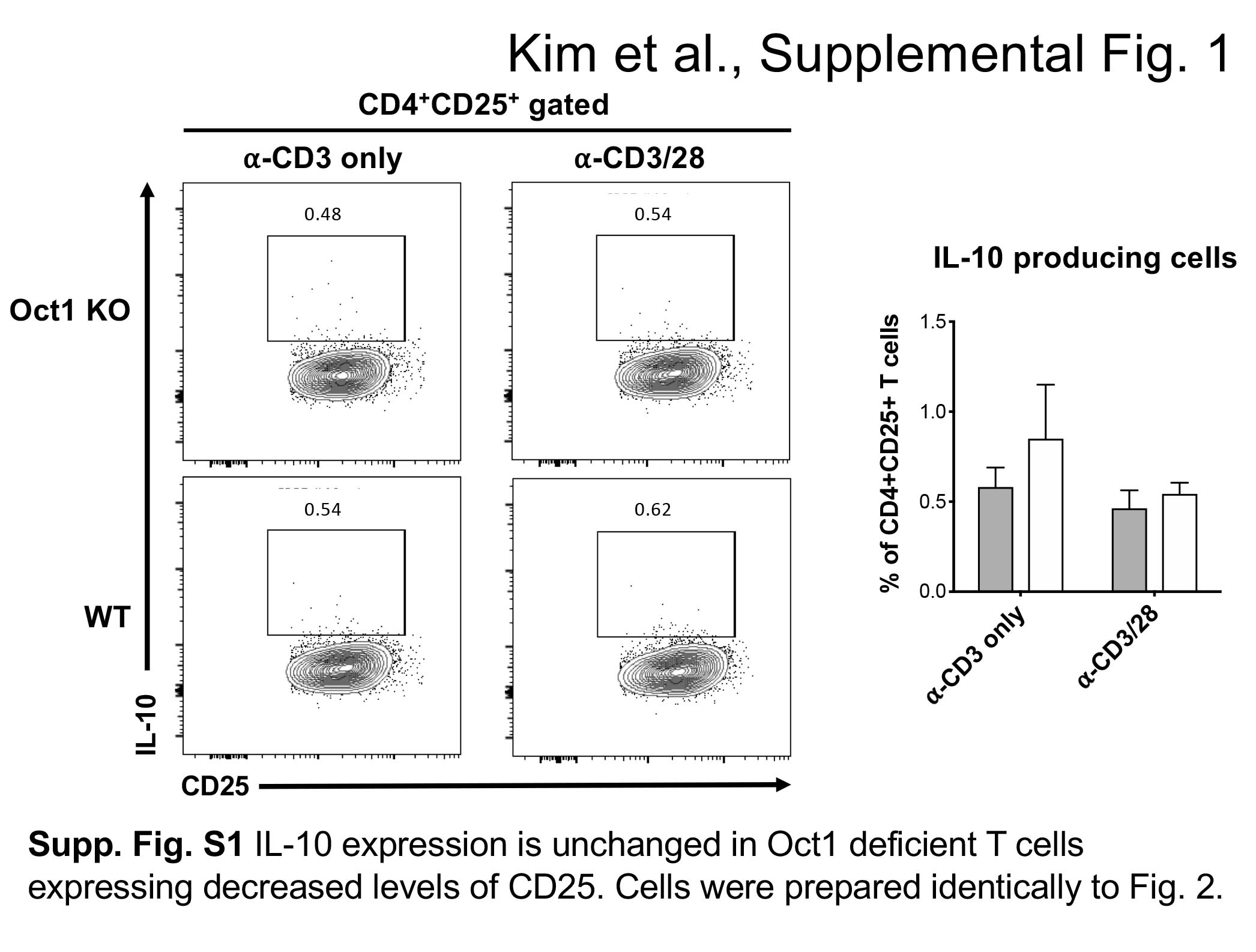

Supplement: Supplementary file 1 — Figure S1 IL-10 expression is unchanged in Oct1-deficient T cells expressing decreased levels of CD25. Cells were prepared identically to Fig. 2. (JPG 239 kb) [file 12974_2019_1523_MOESM1_ESM.jpg]
